# Credit and Loan Approval Classification Using a Bio-Inspired Neural Network

**DOI:** 10.3390/biomimetics9020120

**Published:** 2024-02-17

**Authors:** Spyridon D. Mourtas, Vasilios N. Katsikis, Predrag S. Stanimirović, Lev A. Kazakovtsev

**Affiliations:** 1 Department of Economics, Mathematics-Informatics and Statistics-Econometrics, National and Kapodistrian University of Athens, Sofokleous 1 Street, 10559 Athens, Greece; vaskatsikis@econ.uoa.gr; 2 Laboratory “Hybrid Methods of Modelling and Optimization in Complex Systems”, Siberian Federal University, Prospect Svobodny 79, 660041 Krasnoyarsk, Russia; pecko@pmf.ni.ac.rs (P.S.S.); levk@bk.ru (L.A.K.); 3 Faculty of Sciences and Mathematics, University of Niš, Višegradska 33, 18000 Niš, Serbia; 4 Institute of Informatics and Telecommunications, Reshetnev Siberian State University of Science and Technology, Prospect Krasnoyarskiy Rabochiy 31, 660037 Krasnoyarsk, Russia

**Keywords:** neural networks, Moore-Penrose inverse, weights and structure determination, loan approval classification, beetle antennae search, 15A24, 65F20, 68T05

## Abstract

Numerous people are applying for bank loans as a result of the banking industry’s expansion, but because banks only have a certain amount of assets to lend to, they can only do so to a certain number of applicants. Therefore, the banking industry is very interested in finding ways to reduce the risk factor involved in choosing the safe applicant in order to save lots of bank resources. These days, machine learning greatly reduces the amount of work needed to choose the safe applicant. Taking this into account, a novel weights and structure determination (WASD) neural network has been built to meet the aforementioned two challenges of credit approval and loan approval, as well as to handle the unique characteristics of each. Motivated by the observation that WASD neural networks outperform conventional back-propagation neural networks in terms of sluggish training speed and being stuck in local minima, we created a bio-inspired WASD algorithm for binary classification problems (BWASD) for best adapting to the credit or loan approval model by utilizing the metaheuristic beetle antennae search (BAS) algorithm to improve the learning procedure of the WASD algorithm. Theoretical and experimental study demonstrate superior performance and problem adaptability. Furthermore, we provide a complete MATLAB package to support our experiments together with full implementation and extensive installation instructions.

## 1. Introduction

Since the turn of the century, banks and other financial institutions have been granting loans. Given that credit risk emerges mostly when borrowers are unable or unwilling to pay, rigorous background screening of a customer prior to approval of a loan failing is an essential necessity to sustain oneself in such a business [1,2]. Keep in mind that the amount of non-performing loans in the economy is significant because these loans weigh on bank profits and use valuable resources, limiting banks’ ability to grant new loans [3,4]. Problems in the banking sector can swiftly spread to other sections of the economy, jeopardizing employment and economic growth [5,6]. As a result, there is an urgent need to develop better models for determining whether or not to grant a loan [7,8].

These days, emerging technologies like machine learning and natural language processing greatly reduce the amount of work needed to do such tasks [9,10,11]. Machine learning tasks involving classification are typically found in several fields, such as engineering [12,13], medicine [14], finance and economics [15,16]. Classification presents a significant challenge in these fields.

Neural networks (NNs), which are mostly used for classification and regression challenges, have been effectively implemented in several fields, including medicine, engineering, economics, social science research and finance. In engineering, they are widely used for alloy behavior analysis [17], solar systems measurements [18], and feedback control systems stabilization [19]. Additionally, NNs are frequently used in medical diagnostics to diagnose flat foot [20], diabetic retinopathy [21], and various cancers, such as breast cancer [14] and lung cancer [22]. In contrast, NNs are typically used in the fields of economics and finance for macroeconomic factor prediction [23,24], time series forecasting [25,26], and portfolio optimization [27]. Furthermore, NNs have been effectively used in social science investigation, typically for multiclass classification challenges like classifying occupations [28], assessing the possibility for teleworking in jobs [29], and defining occupational mobility [30].

The primary goal of this work is to create a model for predicting loan acceptance utilizing novel NNs enhanced with state-of-the-art metaheuristic optimization techniques. We will use a feed-forward NN that can handle binary classification tasks in order to achieve this. A training algorithm called weights and structure determination (WASD) will be used in place of the well-known back-propagation approach for training feed-forward NNs. Unlike the back-propagation technique, which iteratively changes the network’s structure, the WASD approach uses the weights direct determination (WDD) procedure to compute the optimal set of weights directly. In the end, this reduces computational complexity by preventing the system from becoming trapped in local minima [31]. Taking into account the multi-input with multi-function activated WASD (MWASD) algorithm for binary classification proposed in [15], the metaheuristic beetle antennae search (BAS) algorithm is paired with the MWASD concept in this work to further improve the performance and structure of the WASD based NNs. In this way, we present a bio-inspired WASD (BWASD) algorithm for binary classification challenges to train a 3-layer feed-forward NN. It is important to note that BAS, which can perform efficient global optimization, has recently gained significant traction in several scientific domains, such as finance [32], robotics [33,34], engineering [35,36], and machine learning [19]. To better address these tasks, BAS has undergone a number of alternations, such as the binary [37] and the semi-integer [38] acceptations. Specifically in machine learning, the WASD and BAS algorithms have been combined in [32] to improve the performance and structure of WASD based NNs for regression-related challenges. Unlike [32], which simply uses the BAS-WASD combination to determine the best structure of the NN in regression-related situations, our approach utilizes BWASD:

1. to identify the ideal structure of the NN;
2. to find the optimal activation function of each hidden layer neuron in binary classification tasks;
3. and to do cross-validation auto-adjustment (i.e., optimize the ratio between the fitting and validation sets).

Results from four experiments demonstrate that the BWASD model outperforms several of the most advanced models of MATLAB’s classification learner in every way.

The primary ideas of this work can be summed up as follows:

- A novel 3-layer feed-forward bio-inspired WASD NN for binary classifications, termed BWASD, is presented.
- The BWASD algorithm merges the BAS and MWASD processes to further improve the performance and structure of the WASD based NNs.
- Taking into account four loan approval datasets, the performance of the MWASD and BWASD models is contrasted.
- Several of the most advanced models of MATLAB’s classification learner are compared with the BWASD model in four experiments.

The structure of the paper is described in the following sections. Section 2 provides an overview of the WDD procedure for binary classification tasks. The 3-layer feed-forward BWASD NN structure, the BWASD algorithm and the whole process for training and the procedure for testing the BWASD NN model are presented in Section 3. Section 4 shows and discusses the findings of four loan approval datasets using the BWASD, the MWASD and several of the most cutting-edge models of MATLAB’s classification learner. Final remarks are provided in Section 5.

## 2. A Novel Weights Direct Determination (WDD) Process for Binary Classification

The WDD process is an essential component of any WASD technique since it eliminates the need for laborious, time-consuming, and frequently erroneous repeating computations to obtain the appropriate weights matching the current hidden layer structure. The WDD approach is claimed to offer reduced computing complexity and speed compared to traditional weight determination methods, while also resolving certain related issues [31]. It is important to note that real numbers are the sole type of input data that the WDD accepts. Prior to being fed into the NN model, the data must additionally be standardized to a range of [−0.5,−0.25]. The NN can manage over-fitting in this way. We can achieve it, if necessary, by using the linear transformation that is illustrated in [26].

In this section, comprehensive explanations of important scientific and theoretical underpinnings are provided in support of the creation of the BWASD NN. But before anything else, it is important to recognize some of the key symbols used in this paper: Transposition is indicated by ${\left(\right)}^{T}$; factorial of η is indicated by η!; pseudoinversion is shown by ${\left(\right)}^{†}$; elementwise exponential is indicated by ${\left(\right)}^{⊙}$; round function is indicated by R(·).

The theorem of the approximation of the Taylor polynomial (ATP) from [39] is restated below.

**Theorem** **1.** *When a target function, Q(·), has the continuous (ρ+1)-order derivative on the range $\left[\lambda_{1},\lambda_{2}\right]$ and ρ is a nonnegative integer, it holds that:*

(1) $$Q\left(\eta \right)=U_{\rho }\left(\eta \right)+V_{\rho }\left(\eta \right),\;\eta \in \left[\lambda_{1},\lambda_{2}\right],$$

*where $V_{\rho }\left(\eta \right)$ and $U_{\rho }\left(\eta \right)$, respectively, imply the error term and ρ-order ATP of Q(η).*

Assume that $Q^{\left(\alpha \right)}\left(\theta \right)$ is the value of the α-order derivative of Q(x) at point θ. The approximation of Q(η) appears below:

(2) $$Q\left(\eta \right)\approx U_{\rho }\left(\eta \right)=\sum_{\alpha =0}^{\rho }\frac{Q^{\left(\alpha \right)}\left(\theta \right)}{\alpha !}{\left(\eta -\theta \right)}^{\alpha },\;\theta \in \left[\lambda_{1},\lambda_{2}\right].$$

**Proposition** **1.** *For approximating multivariable functions, one can apply Theorem 1. Consider $Q(\eta_{1},\eta_{2},\cdots ,\eta_{v})$ be the target function with v variables and continuous (ρ+1)-order partial derivatives in a neighborhood of the origin (0,⋯,0). The ρ-order ATP $U_{\rho }\left(\eta_{1},\eta_{2},\cdots ,\eta_{v}\right)$ about the origin appears below:*

(3) $$U_{\rho }\left(\eta_{1},\eta_{2},\cdots ,\eta_{v}\right)=\sum_{h=0}^{\rho }\sum_{\alpha_{1}+\cdots +\alpha_{v}=h}\frac{\eta_{1}\cdots \eta_{v}}{\alpha_{1}\cdots \alpha_{v}}\left(\frac{\partial^{\alpha_{1}+\cdots +\alpha_{v}}Q\left(0,\cdots ,0\right)}{\partial \eta_{1}^{\alpha_{1}}\cdots \partial \eta_{v}^{\alpha_{v}}}\right),$$

*where $\alpha_{1},\alpha_{2},\cdots ,\alpha_{v}$ are nonnegative integers.*

Consider the input $C=\left[C_{1},C_{2},\cdots ,C_{m}\right]\in R^{1\times m}$ and the target J∈R. The nonlinear function shown next can be utilized to define the relationship between the input variables $C_{1},C_{2},\cdots ,C_{m}$ and the NN’s output target *J*, based on the multi-input NNs described in [31]:

(4) $$Q(C_{1},C_{2},\cdots ,C_{m})=J.$$

The map between the ρ-order ATP $U_{\rho }\left(C_{1},C_{2},\cdots ,C_{m}\right)$ and (4), inline with Proposition 1, appears below:

(5) $$U_{\rho }\left(C_{1},C_{2},\cdots ,C_{m}\right)=\sum_{h=0}^{n-1}k_{h}w_{h},$$

where a power activation function is denoted by $k_{h}=A_{h}\left(C_{1},C_{2},\cdots ,C_{m}\right)\in R^{1\times mn}$; the weight associated with $k_{h}$ is denoted by $w_{h}\in R^{mn}$; and *h* denotes both the number of hidden layer neurons and the power value.

When r∈N samples are taken, the target becomes $J\in R^{r}$ and the input matrix becomes $C=\left[C_{1},C_{2},\cdots ,C_{m}\right]\in R^{r\times m}$, where $C_{j}\in R^{r}$ for j=1,⋯,m. Then, with $k_{r,h}=A_{h}\left(C_{1},C_{2},\cdots ,C_{m}\right)\in R^{r\times mn}$, the weight vector *W* and the input-activation matrix *K* appear below:

(6) $$K=\left[\begin{array}{cccc} k_{1,0} & k_{1,1} & \cdots & k_{1,n-1}\\ k_{2,0} & k_{2,1} & \cdots & k_{2,n-1}\\ \vdots & \vdots & \ddots & \vdots \\ k_{r,0} & k_{r,1} & \cdots & k_{r,n-1} \end{array}\right]\in R^{r\times mn},\;W=\left[\begin{array}{c} w_{0}\\ w_{1}\\ w_{2}\\ \cdots \\ w_{n-1} \end{array}\right]\in R^{mn}.$$

Afterwards, instead of employing the iterative weight training techniques employed in traditional NNs, the weights of the ρ-order ATP NN are created by executing the WDD methodology laid out below [39]:

(7) $$W=K^{†}J.$$

Furthermore, Table 1 presents the four power elementwise activation functions extracted from [26], which are suggested for use in binary classification tasks.

## 3. The Bio-Inspired WASD (BWASD) Model

This section features the 3-layer feed-forward NN structure and the BWASD algorithm.

### 3.1. The Neural Network Structure

**Figure 1:**
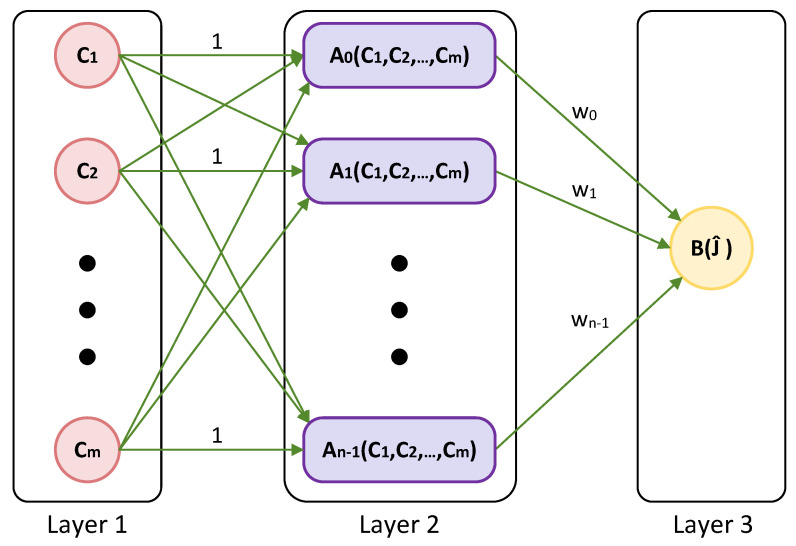
Structure of the BWASD neural network.

Figure 1 illustrates the architecture of the 3-layer feed-forward NN. Specifically, the NN finds the appropriate neuron of Layer 2 with equal weight 1 after receiving the normalized input values $C_{1},C_{2},\cdots ,C_{m}$ from Layer 1 (i.e., the input layer). Note that Layer 2 contains a maximum of *n* active neurons. Moreover, the WDD process is used to acquire the neurons that link Layer 2 and Layer 3 (i.e., the output layer), and these neurons have weights $W_{j},j=1,2,\cdots ,n-1$. Using the following formula, the predictions $\hat{J}$ are computed:

(8) $$\hat{J}=KW.$$

Finally, Layer 3 has a single active neuron that utilizes the elementwise function outlined below:

(9) $$B\left({\hat{J}}_{i}\right)=\left\{\begin{array}{cc} 1 & ,{\hat{J}}_{i}\geq -0.375\\ 0 & ,{\hat{J}}_{i}<-0.375 \end{array},\;\mathrm{for}\;i=1,2,\cdots ,r,\right.$$

where the numbers 0 and 1, respectively, stand for false and true in order to identify something as true or false depending on the related input *C* of the first layer. Also, notice that the number −0.375 is the midpoint of the interval [−0.5,−0.25].

### 3.2. The BWASD Algorithm

**Figure 2:**
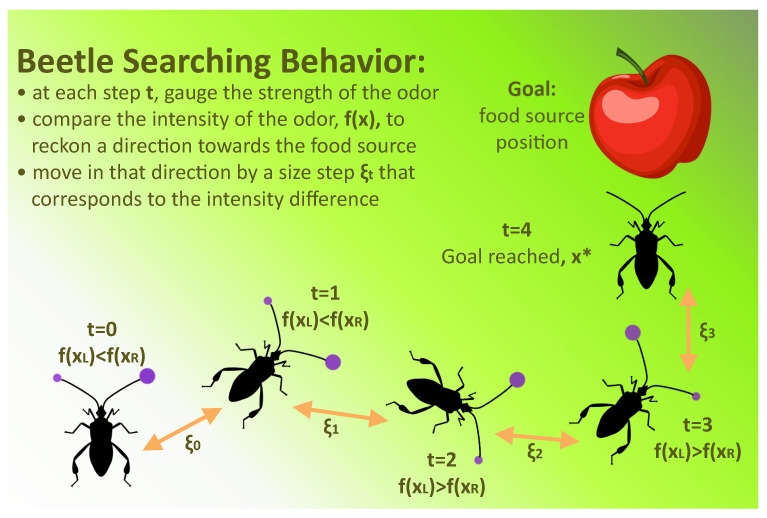
Beetle searching behavior.

The BWASD algorithm, which incorporates the BAS algorithm [40], is responsible for training the NN model. It should be noted that beetles use both of their antennae to search for food, depending on how strong the scent is that they detect on them (Figure 2). This tendency is mimicked by the optimal solution finder of the BAS algorithm, and this approach allows the use of state-of-the-art techniques for optimization (see [41,42,43]). BWASD mimics the behavior of the beetle to find the optimal number of hidden layer neurons in the NN, their power value, and the optimal activation function from Table 1 for each hidden layer neuron. It does this by optimizing the ratio between the fitting and validation sets (i.e., cross-validation auto-adjustment).

First, an objective function must be defined. Consider the training set $X_{tr}\in R^{r\times m}$ with *r* in number samples and their target $J_{tr}\in R^{r}$. The parameter p∈[0.3,0.95]⊆R determines the ratio between the fitting and the validation set. Particularly, the first $r_{1}=pr$ samples of $X_{tr}$ are used for fitting the model and the last $r_{2}=r-r_{1}$ samples for validation. That is, $X_{fi}\in R^{r_{1}\times m}$ is the fitting set and $X_{va}\in R^{r_{2}\times m}$ is the validation set, while $J_{fi}\in R^{r_{1}}$ and $J_{va}\in R^{r_{2}}$ are their target, respectively. Keep in mind that validation aids in ensuring that the model’s success generalizes beyond the training set because it is separate from the fitting set. Then, the *K* matrix is constructed according to Algorithm 1 proposed in [15], which makes use the power activation function in Table 1. For the fitting set $X_{fi}$, the weights of the NN *W* are directly obtained by (7) using $K(1:r_{1})$ and $J_{fi}$. For the validation set $X_{va}$, the NN predictions ${\hat{J}}_{va}$ are obtained by (8) using $K(r_{1}+1:r)$ and *W*, and the mean absolute error (MAE) between the target $J_{va}$ and ${\hat{J}}_{va}$ is gauged via the next formula:

(10) $$E=\frac{1}{r_{2}}\sum_{k=1}^{r_{2}}\left|J_{k}-{\hat{J}}_{k}\right|.$$

It should be noted that the MAE is widely used in machine learning as a loss function for classification challenges, and that it counts errors between paired observations that represent the same situation. Assume the vector $x={\left[p,c,N\right]}^{T}$, where *N* is a vector that includes the hidden layer neurons’ power values and *c* is a vector that contains the numbering of the optimal activation function from Table 1 for each hidden layer neuron. In Algorithm 1, the previously indicated procedure is expressed as an objective function.
**Algorithm 1** Objective function.**Require:** The vector *x*, the input data *X* and the target *J*. 1:**procedure** Ob_func(X,J,x) 2:    Split *x* into *p*, *c* and *N*, and set *r* the rows number of *X*. 3:   Keep only the nonnegative elements in *N* and in *c* only their corresponding activation function numbering. 4:    Calculate the matrix *K* through Algorithm 1 proposed in [15] under the *N* and *c*. 5:    Set $r_{1}=pr$, $r_{2}=r-r_{1}$, $X_{fi}=X\left(1:r_{1},:\right)$, $J_{fi}=J\left(1:r_{1}\right)$, $X_{va}=X\left(r_{1}+1:r,:\right)$ and $J_{va}=J\left(r_{1}+1:r\right)$. 6:    Through the WDD method, calculate *W* utilizing $K(1:r_{1})$ and $J_{fi}$. 7:    Through (8), calculate ${\hat{J}}_{va}$ utilizing $K(r_{1}+1:r)$ and *W*. 8:    Through (10), assign the MAE that was calculated between ${\hat{J}}_{va}$ and $J_{va}$ to *E*. 9:**end procedure****Ensure:** *E*, the error.

Second, by adopting the beetle’s behavior, the objective function in Algorithm 1 is minimized. Consider the vector $x={\left[p,c^{T},N^{T}\right]}^{T}$, where the parameter p∈[0.3,0.95], and *c* is a vector of variable size and its elements take the integer values 1, 2, 3 or 4. These 4 numbers correspond to the activation functions presented in Table 1. Also, the vector *N* has the same size as *c* and its entries take the integer values 0, 1, …, $n_{\max}-1$ or $n_{\max}$. Take note that $n_{\max}$ is the maximum number of hidden layer neurons that the user has set. These $n_{\max}+1$ values represent the power of the activation functions for every neuron in the hidden layer. For instance, $c={\left[2,4\right]}^{T}$ and $N={\left[9,6\right]}^{T}$ indicate the presence of two hidden layer neurons, the first of which operates under the power of 9 using the power sigmoid activation function and the second under the power of 6 using the power softplus activation function.

The beetle’s position is represented by the previously described vector *x* in our method, and the odor concentration at position *x* is represented by the objective function f(x) in Algorithm 1. The minimal value of f(x) acts as a link to the source of the odor. In addition, we use the notation $x^{t}$ with $t=1,2,3,\cdots ,t_{\max}$, where $t_{\max}$ indicates the maximum number of iterations that the user specifies, to denote the position of the beetle at the *t*-th moment. As a result, we set the lower boundary $\mathrm{LB}={\left[0.3,1^{T},0^{T}\right]}^{T}$, where $1,0\in R^{n_{\max}+1}$ denote the all ones and all zeros vectors, respectively, and the upper boundary $\mathrm{UB}={\left[0.9,1^{T}\cdot 4,1^{T}\cdot n_{\max}\right]}^{T}$. In order to guarantee that LB≤x≤UB, the following element-wise function will be used for the element $j=1,\cdots ,2n_{\max}+1$:

(11) $$g\left(x_{j}\right)=\left\{\begin{array}{cc} {\mathrm{UB}}_{j}, & x_{j}>{\mathrm{UB}}_{j}\\ x_{j}, & \mathrm{LB}\leq x_{j}\leq \mathrm{UB}\\ {\mathrm{LB}}_{j}, & x_{j}<{\mathrm{LB}}_{j} \end{array}\right..$$

Thus, a model of searching behavior is defined by the beetle’s chaotic search path in the following manner:

(12) $$h=\frac{\gamma }{\epsilon +∥\gamma ∥},$$

where $\gamma \in R^{2n_{\max}+1}$ implies a random vector of $2n_{\max}+1$ entries and $\epsilon =2^{-52}$. The following formulas are used to create the left $\left(x_{L}\right)$ and right $\left(x_{R}\right)$ antennae, which simulate the beetle’s antennae’s searching behaviors:

(13) $$x_{R}=g\left(R\left(x^{t}+\eta^{t}h\right)\right),\;x_{L}=g\left(R\left(x^{t}-\eta^{t}h\right)\right).$$

where the sensing width of the antennae, $\eta^{t}$, corresponds to the exploit’s capacity at the *t*-th moment. Furthermore, take into account the potential best solution $\left(x_{P}\right)$:

(14) $$x_{P}=g\left(R\left(x^{t}+\xi^{t}\eta^{t}\mathrm{sign}\left(f\left(x_{L}\right)-f\left(x_{R}\right)\right)\right)\right),$$

where the notation $\xi^{t}$ represents a step size that accounts for the rate of convergence after a rise in *t* across the search. Next, the detecting behavior is stated as follows:

(15) $$x^{t+1}=\left\{\begin{array}{cc} x_{P}, & f\left(x_{P}\right)\leq f\left(x^{t}\right)\\ x^{t}, & f\left(x_{P}\right)>f\left(x^{t}\right) \end{array}\right..$$

Finally, the following describes the update rules for η and ξ:

(16) $$\eta^{t+1}=0.991\eta^{i}+0.001,\;\xi^{t+1}=0.991\xi^{i}.$$

It is important to remember that the prerequisites for the previously given technique are as follows:

(17) $$x^{0}={\left[1-q,2-q,\cdots ,2n_{\max}+1-q\right]}^{T},$$

where $q=R(\left(2n_{\max}+1\right)/2)$.

**Figure 3:**
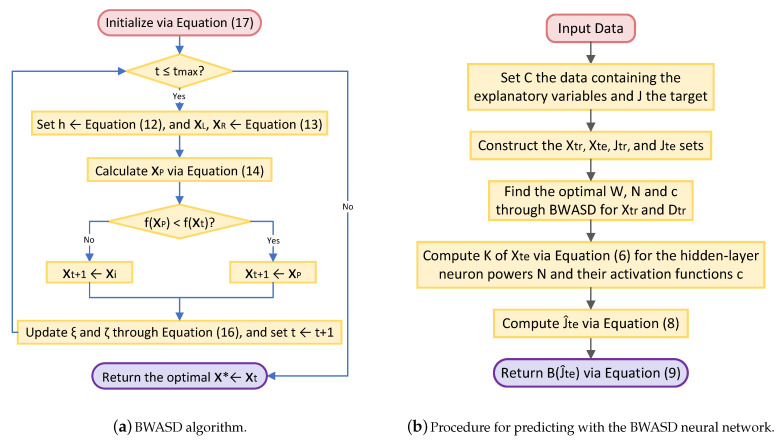
The BWASD algorithm and the procedure for predicting with the BWASD neural network.

After that, on the complete training data set, the BWASD algorithm finds and outputs the optimal: ratio $p^{*}$ between the fitting and validation sets; the optimal *W*; the optimal power value $N^{*}$; and the optimal activation function of each hidden layer neuron $c^{*}$. The full workflow of the BWASD algorithm is illustrated in the diagram of Figure 3a.

Once finding the optimal structure of BWASD NN model of Figure 1, its optimal weights and parameters $p^{*},N^{*},c^{*}$, we set the testing set $X_{te}$ to find the predictions $B({\hat{J}}_{te})$ via (9). The diagram presented in Figure 3b provides an illustration of the comprehensive process for modeling and prediction using the BWASD NN model.

## 4. Experiments

In this section, four datasets are used to conduct four different experiments on credit and loan approval. In these experiments, the performance of the BWASD NN is examined and compared with several top-performing models of MATLAB’s classification learner. The kernel naive Bayes (KNB), fine tree (FTR), linear support vector machine (LSVM), and fine *k*-nearest neighbors (FKNN) are these classification models. The MWASD NN model developed in [15] is also compared because BWASD is an enhanced version of MWASD. For the BWASD model, we have used $\eta^{0}=\xi^{0}=5$, $t_{\max}=21$, and $n_{\max}=10$; for the MWASD model, we have used $n_{\max}=10$ and p=0.8; and for the MATLAB classification models, we have used the default values. It is noteworthy that by clicking the next GitHub link, anyone can obtain the entire development and implementation of the ideas and computation techniques discussed in Section 2, Section 3 and Section 4: https://github.com/SDMourtas/BWASD (accessed on 10 January 2024). Be aware that the MATLAB toolbox includes implementation and installation guidance.

### 4.1. Dataset 1

Customer information entered on an online loan application form is included in the dataset used in this experiment. You may access the dataset, which we will refer to as DA1, by clicking on the provided link: https://www.kaggle.com/datasets/ninzaami/loan-predication?resource=download (accessed on 10 January 2024). It is important to mention that DA1 was provided by a business that wants to automate the real-time loan qualifying process using customer information. DA1 will contain 471 numerical samples under 13 variables when the data preprocessing algorithm described in [15] is used. As a result, the training set is constructed using the first 236 samples, while the testing set is constructed using the final 235 samples.

**Figure 4:**
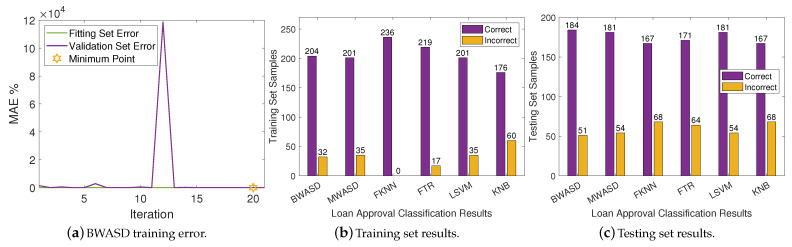
Neural networks results on DA1.

The BWASD training error is shown in Figure 4a, while the NNs classification results for the training and testing sets are displayed in Figure 4b,c, respectively. Figure 4a shows that the validation error is higher than the fitting error, and that the BWASD requires 20 iterations to optimize the NN structure. Particularly, BWASD returned $N^{*}=\left[3,1,3,4\right]$ with $c^{*}=\left[3,2,2,1\right]$ and $p^{*}=0.3$ for the specific run, while MWASD returned $N^{*}=\left[0,1\right]$ with $c^{*}=\left[1,3\right]$. That is, the NN trained under BWASD has 4 hidden layer neurons, while the NN trained under MWASD has 2. Figure 4b shows that FKNN has the best ratio correct/incorrect classifications on the training set, whereas KNB has the worst. Figure 4c shows that BWASD has the best ratio correct/incorrect classifications on the testing set, while FKNN and KNB have the worst.

### 4.2. Dataset 2

The dataset used in this experiment includes results based on credit rating algorithms as well as the possibility that someone may have financial issues in the next two years. It is important to mention that banks ussualy utilize credit scoring algorithms to assess whether to approve a loan based on an estimation of the likelihood of default. You may access the dataset, which we will refer to as DA2, by clicking on the provided link: https://www.kaggle.com/brycecf/give-me-some-credit-dataset?select=cs-training.csv (accessed on 10 January 2024). DA2 will contain 120269 numerical samples under 11 variables when the data preprocessing algorithm described in [15] is used. As a result, the training set is constructed using the first 9179 samples, while the testing set is constructed using the final 111090 samples.

**Figure 5:**
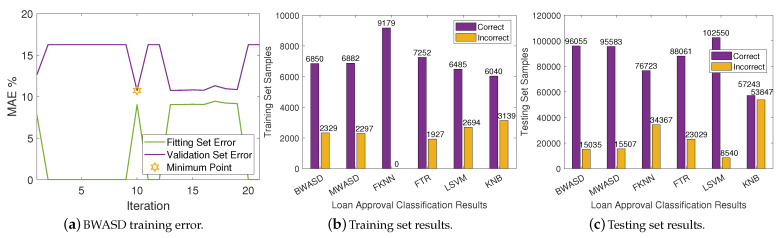
Neural networks results on DA2.

The BWASD training error is shown in Figure 5a, while the NNs classification results for the training and testing sets are displayed in Figure 5b,c, respectively. Figure 5a shows that the validation error is higher than the fitting error, and that the BWASD requires 10 iterations to optimize the NN structure. Particularly, BWASD returned $N^{*}=\left[0,0,3,3,4,6\right]$ with $c^{*}=\left[4,3,3,4,2,3\right]$ and $p^{*}=0.9$ for the specific run, while MWASD returned $N^{*}=\left[0,1,2,3,4\right]$ with $c^{*}=\left[2,3,4,3,4\right]$. That is, the NN trained under BWASD has 6 hidden layer neurons, while the NN trained under MWASD has 5. Figure 5b shows that FKNN has the best ratio correct/incorrect classifications on the training set, whereas KNB has the worst. Figure 5c shows that LSVM has the best ratio correct/incorrect classifications on the testing set and BWASD has the second best, while KNB has the worst.

### 4.3. Dataset 3

Numerous credit card applications are received by commercial banks. Many of them are turned down for a variety of reasons, such as excessive credit record requests, poor income, or large loan balances. Because time is money, manually assessing these applications is tedious, prone to errors, and time-consuming. Fortunately, machine learning can be used to automate this operation, and most commercial banks already do so. The dataset used in this experiment includes results of credit card applications. You may access the dataset, which we will refer to as DA3, by clicking on the provided link: https://www.kaggle.com/datasets/samuelcortinhas/credit-card-approval-clean-data (accessed on 10 January 2024). DA3 will contain 689 numerical samples under 16 variables when the data preprocessing algorithm described in [15] is used. As a result, the training set is constructed using the first 345 samples, while the testing set is constructed using the final 344 samples.

**Figure 6:**
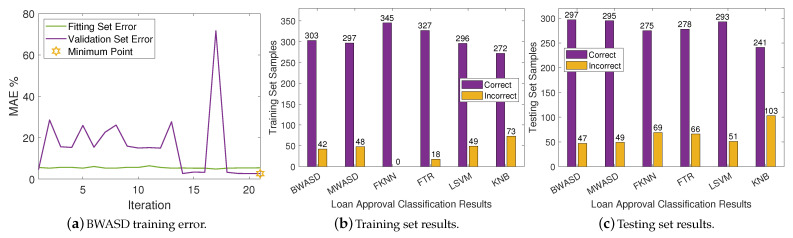
Neural networks results on DA3.

The BWASD training error is shown in Figure 6a, while the NNs classification results for the training and testing sets are displayed in Figure 6b,c, respectively. Figure 6a shows that the validation error is mostly higher than the fitting error, and that the BWASD requires 21 iterations to optimize the NN structure. Particularly, BWASD returned $N^{*}=\left[0,0,2,2,2,5,5\right]$ with $c^{*}=\left[2,1,1,4,4,2,2\right]$ and $p^{*}=0.95$ for the specific run, while MWASD returned $N^{*}=\left[0,1,2\right]$ with $c^{*}=\left[4,3,4\right]$. That is, the NN trained under BWASD has 7 hidden layer neurons, while the NN trained under MWASD has 3. Figure 6b shows that FKNN has the best ratio correct/incorrect classifications on the training set, whereas KNB has the worst. Figure 6c shows that BWASD has the best ratio correct/incorrect classifications on the testing set, while KNB has the worst.

### 4.4. Dataset 4

Banks require decision-making guidelines regarding which loans they will approve or deny in order to reduce their own losses. Loan managers take into account an applicant’s socioeconomic and demographic profiles before making a determination about the loan application. The dataset used in this experiment includes results of loan applications based on applicants socioeconomic and demographic profiles. You may access the dataset, which we will refer to as DA4, by clicking on the provided link: https://www.kaggle.com/datasets/mpwolke/cusersmarildownloadsgermancsv (accessed on 10 January 2024). DA4 will contain 999 numerical samples under 20 variables when the data preprocessing algorithm described in [15] is used. As a result, the training set is constructed using the first 500 samples, while the testing set is constructed using the final 499 samples.

**Figure 7:**
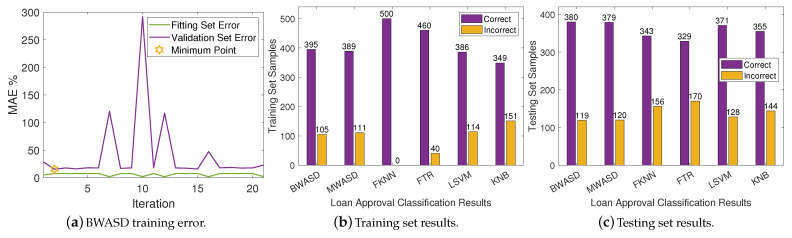
Neural networks results on DA4.

The BWASD training error is shown in Figure 7a, while the NNs classification results for the training and testing sets are displayed in Figure 7b,c, respectively. Figure 7a shows that the validation error is higher than the fitting error, and that the BWASD requires 2 iterations to optimize the NN structure. Particularly, BWASD returned $N^{*}=\left[0,2,3,3,2,4\right]$ with $c^{*}=\left[4,1,1,1,1,4\right]$ and $p^{*}=0.95$ for the specific run, while MWASD returned $N^{*}=\left[0,1,2\right]$ with $c^{*}=\left[4,3,3\right]$. That is, the NN trained under BWASD has 6 hidden layer neurons, while the NN trained under MWASD has 3. Figure 7b shows that FKNN has the best ratio correct/incorrect classifications on the training set, whereas KNB has the worst. Figure 7c shows that BWASD has the best ratio correct/incorrect classifications on the testing set, while FTR has the worst.

### 4.5. Performance Measures and Discussion

The models statistics for DA1-DA4 on the testing set are shown in Table 2, Table 3, Table 4 and Table 5, correspondingly. The MAE, true positive (TP), true negative (TN), false positive (FP), false negative (FN), precision, recal, accuracy and F-score are the performance gauges considered in this analysis. Consult [44] for further information and a detailed examination of these gauges. Additionally, the accuracy of the classification models is statistically evaluated using the mid-p-value McNemar test in Table 6, Table 7 and Table 8.

In Table 2, BWASD appears to have the finest MAE, accuracy and F-score, and the second finest TP, FP, precision and recal. FTR has the best TN, FN and recal, and the worst TP, FP, and precision. The results of MWASD and LSVM are identical and they have the best TP, FP, and precision. Additionally, KNB has the worst MAE, TN, FN, recal, accuracy and F-score. According to the aforementioned statistics, the performance of BWASD is the best, while KNB is the poorest.

In Table 3, LSVM appears to have the finest MAE, TN, FN, recal and accuracy, whereas KNB has finest TP, FP and precision, and FTR has the finest F-score. BWASD has the second best MAE, TN, FN, recal and accuracy, but BWASD has better TP, FP, precision and F-score than LSVM. Additionally, KNB has the worst MAE, TN, FN, recal and accuracy, whereas LSVM has the worst TP, FP, precision and F-score. According to the aforementioned statistics, the performance of BWASD is the best overall, while KNB is the poorest.

In Table 4, BWASD appears to have the finest MAE, TN, FN, recal, accuracy and F-score. LSVM has the finest TP, FP and precision. The results of MWASD and LSVM are identical and they have the best TP, FP, and precision. Additionally, KNB has the worst statistic measurements, FKNN has the second worst MAE, TP, FP, precision, recal, accuracy and F-score, whereas LSVM has the second worst TN and FN. According to the aforementioned statistics, the performance of BWASD is the best, while KNB is the poorest.

In Table 5, BWASD appears to have the finest MAE, accuracy and F-score, the second finest recall, and the third finest TP, FP, TN, FN and precision. KNB has the best TP, FP and precision, FTR has the best TN and FN, and MWASD has the best recal. LSVM has the best TP, FP and precision. Additionally, FTR has the worst MAE, TP, FP, precision, accuracy and F-score, whereas KNB has the worst TN, FN and recal. According to the aforementioned statistics, the performance of BWASD is the best, while FTR is the poorest.

The BWASD model is compared to all other models in Table 6, Table 7 and Table 8 using the mid-p-value McNemar test to statistically evaluate the classification models’ accuracies. The McNemar test is a form of homogeneity test that applies to contingency tables and is a distribution-free statistical hypothesis test. The test determines whether the binary classification models’ accuracies differ or whether one binary classification model outperforms the other. We perform the McNemar test specifically using the MATLAB function testcholdout, as described in [45,46]. It is important to note that the simulation experiments in [45,46,47] show that this test has good statistical power and achieves nominal coverage. The statistical analysis in this subsection follows the recommendations in [47]. According to the marginal homogeneity null hypothesis, each outcome’s two marginal probabilities are equal. In our investigation, the null hypothesis claims that the accuracy of the predicted class labels from the NN model Z and the BWASD is equal, where Z refers to MWASD, FKNN, FTR, LSVM or KNB. Additionally, we consider the following three alternative hypothesis (AH):

- AH1: For predicting the class labels, the NN model Z and the BWASD have unequal accuracies.
- AH2: For predicting the class labels, the NN model Z is more accurate than the BWASD.
- AH3: For predicting the class labels, the NN model Z is less accurate than the BWASD.

In this way, we conduct three McNemar tests under three different alternative hypothesis to assess. Each test determines whether to reject or not to reject the null hypothesis at the 5% significance level. Keep in mind that an outcome is considered statistically significant if it allows us to reject the null hypothesis, and that lower *p*-values (usually ≤ 0.05) are seen as more convincing proof to reject the null hypothesis.

Table 6 shows the McNemar’s test results for AH1. In DA1, DA3 and DA4, when comparing BWASD to Z = {FKNN, FTR, KNB}, a *p*-value of almost zero from the McNemar test indicates that there is enough proof to reject the null hypothesis. In other words, the predicted accuracies of the Z and BWASD models are not equal. On the other hand, when comparing BWASD to Z = {MWASD, LSVM}, a *p*-value that is far from zero indicates that there is enough proof to not reject the null hypothesis. In other words, the predicted accuracies of the Z and BWASD models are equal. In DA2, when comparing BWASD to Z = {MWASD, FKNN, FTR, LSVM, KNB}, a *p*-value of almost zero indicates that there is enough proof to reject the null hypothesis. In other words, the predicted accuracies of the Z and BWASD models are not equal.

Table 7 shows the McNemar’s test results for AH2. In DA1, DA3 and DA4, when comparing BWASD to Z = {MWASD, FKNN, FTR, KNB, LSVM}, a *p*-value of one or almost one from the McNemar test indicates that there is not enough proof to reject the null hypothesis. In other words, the predicted accuracies of the Z and BWASD models are equal. In DA2, when comparing BWASD to Z = {MWASD, FKNN, FTR, KNB}, a *p*-value of one or almost one indicates that there is not enough proof to reject the null hypothesis. In other words, the predicted accuracies of the Z and BWASD models are equal. However, when comparing BWASD to Z = {LSVM}, a *p*-value of zero indicates that there is enough proof to reject the null hypothesis. In other words, the Z model is more accurate than the BWASD.

Table 8 shows the McNemar’s test results for AH3. In DA1, DA3 and DA4, when comparing BWASD to Z = {FKNN, FTR, KNB}, a *p*-value of almost zero from the McNemar test indicates that there is enough proof to reject the null hypothesis. In other words, the Z model is less accurate than the BWASD. On the other hand, when comparing BWASD to Z = {MWASD, LSVM}, a *p*-value that is far from zero indicates that there is enough proof to not reject the null hypothesis. In other words, the predicted accuracies of the Z and BWASD models are equal. In DA2, when comparing BWASD to Z = {MWASD, FKNN, FTR, KNB}, a *p*-value of almost zero indicates that there is enough proof to reject the null hypothesis. In other words, the Z model is less accurate than the BWASD. On the other hand, when comparing BWASD to Z = {LSVM}, a *p*-value of one indicates that there is enough proof to not reject the null hypothesis. In other words, the predicted accuracies of the Z and BWASD models are equal.

Therefore, based on Table 2, Table 3, Table 4, Table 5, Table 6, Table 7 and Table 8 statistics, we conclude that the BWASD is the best performing model in DA1-DA4. In broad terms, the BWASD model consistently provided great results in the classification of loan approval tasks, and it performs rather well when compared to traditional NN models. Therefore, the BWASD model can be beneficial for various businesses. These include businesses looking to automate the evaluation of loan applications based on customer information, banks evaluating credit card applications, banks evaluating loan applications based on an estimation of the likelihood of default, and banks evaluating loan applications based on the socioeconomic and demographic profiles of applicants.

## 5. Conclusions

This work presents a bio-inspired WASD NN for binary classification challenges, named BWASD. The findings of experiments in four loan approval datasets demonstrate that the BWASD model performs better than the MWASD model and several cutting-edge models of MATLAB’s classification learner. Therefore, the BWASD model has shown to be an excellent stand-in for determining whether or not to approve a loan. It is significant to note that the BWASD NN model can only be trained and tested using actual numerical data as input due to restrictions imposed by the WDD method. Future research will therefore focus on properly adjusting and applying it to other binary classification challenges across multiple scientific disciplines.

In this context, the BWASD model could be modified for use in the engineering domain to analyze alloy behavior or data from solar systems, as shown in [17,18]. The BWASD model could also be adjusted for use in the medicine domain to analyze diagnostic data, as demonstrated in [21]. Finally, integrating BAS alternations, like [37,38], may help to improve the accuracy of the BWASD model even further.

## Figures and Tables

**Table 1:** Options of power activation functions.

Name	$A_{h}\left(X\right)$	Range	Numbering
**Power**	$X^{h}$	(−∞,∞)	1
**Power sigmoid**	$\frac{e^{X^{h}}}{e^{X^{h}}+1}$	$\left[\frac{1}{2},1\right)$	2
**Power inverse exponential**	$e^{-X^{h}}$	(0,1)	3
**Power softplus**	$\ln(1+e^{X^{h}})$	(0,∞)	4

**Table 2:** Neural network models’ statistics in DA1.

DA1	Neural Network Models					
Statistic	BWASD	MWASD	FKNN	FTR	LSVM	KNB
**MAE**	0.2170	0.2297	0.2893	0.2723	0.2297	0.2893
**FP**	0.0304	0.0243	0.1646	0.1707	0.0243	0.0243
**TP**	0.9695	0.9756	0.8353	0.8292	0.9756	0.9756
**FN**	0.6478	0.7042	0.5774	0.5070	0.7042	0.9014
**TN**	0.3521	0.2957	0.4225	0.4929	0.2957	0.0985
**Precision**	0.9695	0.9756	0.8353	0.8292	0.9756	0.9756
**Recal**	0.5994	0.5807	0.5912	0.6205	0.5807	0.5197
**Accuracy**	0.7829	0.7702	0.7106	0.7276	0.7702	0.7106
**F-score**	0.7408	0.7281	0.6924	0.7098	0.7281	0.6782

**Table 3:** Neural network models’ statistics in DA2.

DA2	Neural Network Models					
Statistic	BWASD	MWASD	FKNN	FTR	LSVM	KNB
**MAE**	0.1353	0.1395	0.3093	0.2073	0.0768	0.4847
**FP**	0.4028	0.3958	0.4669	0.2932	0.5718	0.1747
**TP**	0.5971	0.6041	0.5330	0.7067	0.4281	0.8252
**FN**	0.1248	0.1295	0.3032	0.2039	0.0575	0.4968
**TN**	0.8751	0.8704	0.6967	0.7960	0.9424	0.5031
**Precision**	0.5971	0.6041	0.5330	0.7067	0.4281	0.8252
**Recal**	0.8270	0.8233	0.6374	0.7760	0.8815	0.6242
**Accuracy**	0.8646	0.8604	0.6906	0.7926	0.9231	0.5152
**F-score**	0.6935	0.6969	0.5805	0.7398	0.5764	0.7107

**Table 4:** Neural network models’ statistics in DA3.

DA3	Neural Network Models					
Statistic	BWASD	MWASD	FKNN	FTR	LSVM	KNB
**MAE**	0.1366	0.1424	0.2005	0.1918	0.1482	0.2994
**FP**	0.0915	0.0784	0.2091	0.1895	0.0718	0.3856
**TP**	0.9084	0.9215	0.7908	0.8104	0.9281	0.6143
**FN**	0.1727	0.1937	0.1937	0.1937	0.2094	0.2303
**TN**	0.8272	0.8062	0.8062	0.8062	0.7905	0.7696
**Precision**	0.9084	0.9215	0.7908	0.8104	0.9281	0.6143
**Recal**	0.8402	0.8263	0.8032	0.8070	0.8158	0.7272
**Accuracy**	0.8633	0.8575	0.7994	0.8081	0.8517	0.7005
**F-score**	0.8730	0.8713	0.7969	0.8087	0.8683	0.6660

**Table 5:** Neural network models’ statistics in DA4.

DA4	Neural Network Models					
Statistic	BWASD	MWASD	FKNN	FTR	LSVM	KNB
**MAE**	0.2384	0.2404	0.3126	0.3406	0.2565	0.2885
**FP**	0.1016	0.1101	0.1949	0.2768	0.0960	0.0169
**TP**	0.8983	0.8898	0.8050	0.7231	0.9039	0.9830
**FN**	0.5724	0.5586	0.6000	0.4965	0.6482	0.9517
**TN**	0.4275	0.4413	0.4000	0.5034	0.3517	0.0482
**Precision**	0.8983	0.8898	0.8050	0.7231	0.9039	0.9830
**Recal**	0.6107	0.6143	0.5729	0.5928	0.5823	0.5080
**Accuracy**	0.7615	0.7595	0.6873	0.6593	0.7434	0.7114
**F-score**	0.7271	0.7268	0.6694	0.6515	0.7083	0.6699

**Table 6:** McNemar’s test results for AH1.

BWASD	DA1		DA2	
vs	Null Hypothesis	*p*-Value	Null Hypothesis	*p*-Value
**MWASD**	not rejected	0.2187	rejected	0
**FKNN**	rejected	0.0050	rejected	0
**FTR**	rejected	0.0288	rejected	0
**KNB**	rejected	0.0009	rejected	0
**LSVM**	not rejected	0.2187	rejected	0
**BWASD**	**DA3**		**DA4**	
**vs**	**Null Hypothesis**	***p*** **-Value**	**Null Hypothesis**	***p*** **-Value**
**MWASD**	not rejected	0.5488	not rejected	0.8600
**FKNN**	rejected	0.0021	rejected	0.0006
**FTR**	rejected	0.0090	rejected	$10^{-6}$
**KNB**	rejected	$10^{-7}$	rejected	0.0095
**LSVM**	not rejected	0.2668	not rejected	0.1741

**Table 7:** McNemar’s test results for AH2.

BWASD	DA1		DA2	
vs	Null Hypothesis	*p*-Value	Null Hypothesis	*p*-Value
**MWASD**	not rejected	0.8906	not rejected	1
**FKNN**	not rejected	0.9975	not rejected	1
**FTR**	not rejected	0.9856	not rejected	1
**KNB**	not rejected	0.9995	not rejected	1
**LSVM**	not rejected	0.8906	rejected	0
**BWASD**	**DA3**		**DA4**	
**vs**	**Null Hypothesis**	***p*** **-Value**	**Null Hypothesis**	***p*** **-Value**
**MWASD**	not rejected	0.7256	not rejected	0.5700
**FKNN**	not rejected	0.9989	not rejected	0.9997
**FTR**	not rejected	0.9955	not rejected	1
**KNB**	not rejected	1	not rejected	0.9952
**LSVM**	not rejected	0.8666	not rejected	0.9129

**Table 8:** McNemar’s test results for AH3.

BWASD	DA1		DA2	
vs	Null Hypothesis	*p*-Value	Null Hypothesis	*p*-Value
**MWASD**	not rejected	0.1094	rejected	0
**FKNN**	rejected	0.0025	rejected	0
**FTR**	rejected	0.0144	rejected	0
**KNB**	rejected	0.0004	rejected	0
**LSVM**	not rejected	0.1094	not rejected	1
**BWASD**	**DA3**		**DA4**	
**vs**	**Null Hypothesis**	***p*** **-Value**	**Null Hypothesis**	***p*** **-Value**
**MWASD**	not rejected	0.2744	not rejected	0.4300
**FKNN**	rejected	0.0011	rejected	0.0003
**FTR**	rejected	0.0045	rejected	2 $\times \;10^{-6}$
**KNB**	rejected	5 $\times \;10^{-8}$	rejected	0.0048
**LSVM**	not rejected	0.1334	not rejected	0.0871

## Data Availability

Publicly available datasets were analyzed in this study. This data can be found here: https://www.kaggle.com/datasets/ninzaami/loan-predication?resource=download (accessed on 10 January 2024); https://www.kaggle.com/brycecf/give-me-some-credit-dataset?select=cs-training.csv (accessed on 10 January 2024); https://www.kaggle.com/datasets/samuelcortinhas/credit-card-approval-clean-data (accessed on 10 January 2024); https://www.kaggle.com/datasets/mpwolke/cusersmarildownloadsgermancsv (accessed on 10 January 2024).

## References

[1] Kesraoui A., Lachaab M., Omri A. (2022). The impact of credit risk and liquidity risk on bank margins during economic fluctuations: Evidence from MENA countries with a dual banking system. Appl. Econ..

[2] Li Z., Liang S., Pan X., Pang M. (2024). Credit risk prediction based on loan profit: Evidence from Chinese SMEs. Res. Int. Bus. Financ..

[3] Naili M., Lahrichi Y. (2022). The determinants of banks’ credit risk: Review of the literature and future research agenda. Int. J. Financ. Econ..

[4] Zhang X., Yu L. (2024). Consumer credit risk assessment: A review from the state-of-the-art classification algorithms, data traits, and learning methods. Expert Syst. Appl..

[5] Abdelaziz H., Rim B., Helmi H. (2022). The interactional relationships between credit risk, liquidity risk and bank profitability in MENA region. Glob. Bus. Rev..

[6] Huang Y., Li Z., Qiu H., Tao S., Wang X., Zhang L. (2023). BigTech credit risk assessment for SMEs. China Econ. Rev..

[7] Bhatore S., Mohan L., Reddy Y.R. (2020). Machine learning techniques for credit risk evaluation: A systematic literature review. J. Bank. Financ. Technol..

[8] Pang M., Li Z. (2024). A novel profit-based validity index approach for feature selection in credit risk prediction. AIMS Math..

[9] Singh V., Yadav A., Awasthi R., Partheeban G.N. Prediction of modernized loan approval system based on machine learning approach. Proceedings of the 2021 International Conference on Intelligent Technologies (CONIT).

[10] Lohani B.P., Trivedi M., Singh R.J., Bibhu V., Ranjan S., Kushwaha P.K. Machine learning based model for prediction of loan approval. Proceedings of the 2022 3rd International Conference on Intelligent Engineering and Management (ICIEM).

[11] Weng C., Huang C. (2021). A hybrid machine learning model for credit approval. Appl. Artif. Intell..

[12] Bigdeli B., Pahlavani P., Amirkolaee H.A. (2021). An ensemble deep learning method as data fusion system for remote sensing multisensor classification. Appl. Soft Comput..

[13] Sun Y., Zhang J., Li G., Wang Y., Sun J., Jiang C. (2019). Optimized neural network using beetle antennae search for predicting the unconfined compressive strength of jet grouting coalcretes. Int. J. Numer. Anal. Methods Geomech..

[14] Raj R.J.S., Shobana S.J., Pustokhina I.V., Pustokhin D.A., Gupta D., Shankar K. (2020). Optimal feature selection-based medical image classification using deep learning model in internet of medical things. IEEE Access.

[15] Simos T.E., Katsikis V.N., Mourtas S.D. (2022). A multi-input with multi-function activated weights and structure determination neuronet for classification problems and applications in firm fraud and loan approval. Appl. Soft Comput..

[16] Zeng T., Zhang Y., Li Z., Qiu B., Ye C. (2020). Predictions of USA presidential parties from 2021 to 2037 using historical data through square wave-activated WASD neural network. IEEE Access.

[17] Huang C., Jia X., Zhang Z. (2018). A modified back propagation artificial neural network model based on genetic algorithm to predict the flow behavior of 5754 aluminum alloy. Materials.

[18] Premalatha N., Arasu A.V. (2016). Prediction of solar radiation for solar systems by using ANN models with different back propagation algorithms. J. Appl. Res. Technol..

[19] Mourtas S.D., Katsikis V.N., Kasimis C. (2022). Feedback control systems stabilization using a bio-inspired neural network. EAI Endorsed Trans. AI Robot..

[20] Chen L., Huang Z., Li Y., Zeng N., Liu M., Peng A., Jin L. (2019). Weight and structure determination neural network aided with double pseudoinversion for diagnosis of flat foot. IEEE Access.

[21] Gayathri S., Krishna A.K., Gopi V.P., Palanisamy P. (2020). Automated binary and multiclass classification of diabetic retinopathy using Haralick and multiresolution features. IEEE Access.

[22] Daliri M.R. (2012). A hybrid automatic system for the diagnosis of lung cancer based on genetic algorithm and fuzzy extreme learning machines. J. Med Syst..

[23] Zhang Y., Guo D., Luo Z., Zhai K., Tan H. (2016). CP-activated WASD neuronet approach to Asian population prediction with abundant experimental verification. Neurocomputing.

[24] Zhang Y., Xue Z., Xiao M., Ling Y., Ye C. (2017). Ten-quarter projection for Spanish central government debt via WASD neuronet. Proceedings of the International Conference on Neural Information Processing.

[25] Mourtas S.D. (2022). A weights direct determination neuronet for time-series with applications in the industrial indices of the federal reserve bank of St. Louis. J. Forecast..

[26] Mourtas S.D., Drakonakis E., Bragoudakis Z. (2023). Forecasting the gross domestic product using a weight direct determination neural network. AIMS Math..

[27] Leung M.F., Wang J. (2022). Cardinality-constrained portfolio selection based on collaborative neurodynamic optimization. Neural Networks.

[28] Matbouli Y.T., Alghamdi S.M. (2022). Statistical machine learning regression models for salary prediction featuring economy wide activities and occupations. Information.

[29] Generalao I.N. (2021). Measuring the telework potential of jobs: Evidence from the international standard classification of occupations. Philipp. Rev. Econ..

[30] Groes F., Kircher P., Manovskii I. (2015). The U-shapes of occupational mobility. Rev. Econ. Stud..

[31] Zhang Y., Chen D., Ye C. (2019). Deep Neural Networks: WASD Neuronet Models, Algorithms, and Applications.

[32] Simos T.E., Katsikis V.N., Mourtas S.D. (2022). Multi-input bio-inspired weights and structure determination neuronet with applications in European Central Bank publications. Math. Comput. Simul..

[33] Cheng Y., Li C., Li S., Li Z. (2020). Motion planning of redundant manipulator with variable joint velocity limit based on beetle antennae search algorithm. IEEE Access.

[34] Fan Y., Shao J., Sun G. (2019). Optimized PID controller based on beetle antennae search algorithm for electro-hydraulic position servo control system. Sensors.

[35] Li X., Jiang H., Niu M., Wang R. (2020). An enhanced selective ensemble deep learning method for rolling bearing fault diagnosis with beetle antennae search algorithm. Mech. Syst. Signal Process..

[36] Li X., Zang Z., Shen F., Sun Y. (2020). Task offloading scheme based on improved contract net protocol and beetle antennae search algorithm in fog computing networks. Mobile Netw. Appl..

[37] Mourtas S.D., Katsikis V.N. (2021). V-Shaped BAS: Applications on large portfolios selection problem. Comput. Econ..

[38] Katsikis V.N., Mourtas S.D. (2021). Diversification of time-varying tangency portfolio under nonlinear constraints through semi-integer beetle antennae search algorithm. AppliedMath.

[39] Zhang Y., Yu X., Xiao L., Li W., Fan Z., Zhang W. (2013). Weights and structure determination of articial neuronets. Self-Organization: Theories and Methods.

[40] Jiang X., Li S. (2017). BAS: Beetle antennae search algorithm for optimization problems. arXiv.

[41] Zhu Z., Zhang Z., Man W., Tong X., Qiu J., Li F. A new beetle antennae search algorithm for multi-objective energy management in microgrid. Proceedings of the 13th IEEE Conf. Industrial Electronics and Applications (ICIEA).

[42] Wu Q., Shen X., Jin Y., Chen Z., Li S., Khan A.H., Chen D. (2019). Intelligent beetle antennae search for UAV sensing and avoidance of obstacles. Sensors.

[43] Xu X., Deng K., Shen B. (2020). A beetle antennae search algorithm based on Lévy flights and adaptive strategy. Syst. Sci. Control Eng..

[44] Tharwat A. (2020). Classification assessment methods. Appl. Comput. Inform..

[45] Kucer M., Loui A.C., Messinger D.W. (2018). Leveraging expert feature knowledge for predicting image aesthetics. IEEE Trans. Image Process..

[46] Zhou T., Liu M., Thung K.H., Shen D. (2019). Latent representation learning for Alzheimer’s disease diagnosis with incomplete multi-modality neuroimaging and genetic data. IEEE Trans. Med Imaging.

[47] Fagerland M.W., Lydersen S., Laake P. (2013). The McNemar test for binary matched-pairs data: Mid-p and asymptotic are better than exact conditional. BMC Med Res. Methodol..

